# Post-procedural elevated cardiac troponin I and the association with 5-year mortality in patients undergoing elective PCI

**DOI:** 10.1016/j.heliyon.2024.e27979

**Published:** 2024-03-22

**Authors:** Queyun Sun, Pei Zhu, Jingjing Xu, Lin Jiang, Yan Chen, Xueyan Zhao, Lei Song, Yuejin Yang, Runlin Gao, Bo Xu, Jinqing Yuan, Ying Song

**Affiliations:** Fu Wai Hospital, National Center for Cardiovascular Diseases, Chinese Academy of Medical Sciences, A 167 Beilishi Road, Xicheng District, Beijing, 100037, China

## Abstract

**Background:**

The clinically meaningful cardiac troponin I (cTnI) threshold associated with the long-term prognosis in patients undergoing elective percutaneous coronary intervention (PCI) is still debated.

**Objective:**

To assess the association between different thresholds for post-procedural cTnI and 5-year mortality.

**Methods:**

The study included 4059 consecutive patients with normal baseline cTnI values who underwent elective PCI. The post-procedural cTnI level was measured at 8–48 h after PCI. The main study endpoints were 5-year all-cause mortality and cardiovascular mortality.

**Results:**

A cTnI ≥5 times the upper reference limit (URL) as defined by the fourth universal definition of myocardial infarction (4th UDMI), ≥35 times as defined by the Academic Research Consortium-2 criteria, and ≥70 times as defined by the Society for Cardiovascular Angiography and Interventions (SCAI [2014]) was identified in 33%, 6.6%, and 3.3% of patients, respectively. During 5 years of follow-up, the all-cause mortality rate was 3.4% (n = 132) and the cardiovascular mortality rate was 2.0% (n = 77). Both all-cause mortality and cardiovascular mortality increased with higher peak cTnI, and were independently predicted by a cTnI ≥70 times the URL (adjusted hazard ratio [HR] 2.45, 95% confidence interval [CI] 1.20–5.02 and adjusted HR 3.17, 95% CI 1.31–7.67, respectively; reference, cTnI <1 × URL]. The SCAI (2014) threshold was significantly associated with 5-year cardiovascular mortality (adjusted HR 2.66, 95% CI 1.20–5.89; reference, cTnI, <70 × URL) and all-cause mortality (adjusted HR 2.23, 95% CI 1.16–4.30; reference, cTnI <70 × URL).

**Conclusion:**

In patients with normal pre-procedural cTnI who underwent elective PCI, a post-procedural cTnI ≥70 times the URL independently predicted 5-year all-cause and cardiovascular mortality. Therefore, only the SCAI (2014) post-procedural cTnI threshold was independently associated with long-term mortality.

## 1 Introduction

Percutaneous coronary intervention (PCI) is an effective method for coronary revascularization. However, in clinical practice, periprocedural myocardial infarction (PMI) sometimes occurs during the PCI procedure [1] because of restricted coronary artery flow [2], side branch occlusion [3], or an impaired microcirculation [4]. PMI is associated with an increased major adverse cardiovascular event rate and has a significant impact on the long-term prognosis [1,5]. Many studies have investigated the diagnostic criteria for PMI and their prognostic impact. The main diagnostic criteria for PMI are myocardial biomarker levels. However, the various definitions use different myocardial biomarker thresholds, which may have different prognostic implications [6,7]. Compared with other myocardial biomarkers commonly used in the clinical setting, cardiac troponin I (cTnI) has better myocardial specificity than creatine kinase (CK)-MB. Furthermore, high-sensitivity troponin I, although able to detect myocardial injury earlier owing to its high sensitivity, may lead to an excessive number of false-positive results because patients often have minor myocardial injuries as a result of surgical stress, anaesthesia, and other complications. It is now generally accepted that an increased cTnI level is associated with more severe myocardial injury, thus providing a clearer indication in the clinical setting. When other evidence of myocardial ischemia necessary for a diagnosis of PMI is missing (Supplementary Table 1), we can simply use the different cTnI thresholds in the different definitions, which represent different degrees of myocardial injury, and select the best threshold for clinical diagnosis. The aim of this study was to investigate the relationship between peak troponin levels within 48 h after PCI and long-term mortality in the hope of providing a reference for selection of more appropriate thresholds for PMI.

## 2 Patients and methods

### 2.1 Study population

The data analyzed in this single-center retrospective cohort study were collected from consecutive patients who underwent PCI between January 2013 and December 2013 at Fu Wai Hospital, National Center for Cardiovascular Diseases in Beijing, China.

### 2.2 PCI procedure and medications

The PCI strategy and type of stent used were left to the discretion of the treating physician. Patients undergoing selective PCI who were not on long-term aspirin and P2Y12 inhibitor therapy received oral aspirin 300 mg and a loading dose of clopidogrel 300 mg or ticagrelor 180 mg at least 24 h before the procedure. All patients received unfractionated heparin 100 U/kg during the procedure, and use of glycoprotein IIb/IIIa inhibitors was at the discretion of the operator. After the procedure, aspirin 100 mg/day was prescribed indefinitely, with clopidogrel 75 mg/day or ticagrelor 90 mg twice daily recommended for at least 1 year after PCI.

### 2.3 Measurement of biomarkers and selection of thresholds

The cTnI level was measured using conventional methods. Blood samples for cTnI were collected routinely before PCI, 8–24 h after PCI, and daily thereafter. More frequent measurements were taken to assess post-PCI peak cTnI if the post-procedural cTnI level was elevated or if complications were noted. cTnI levels were measured using the Access AccuTnI assay with the Access 2 chemiluminescent immunoassay system (Beckman Coulter, Brea, CA, USA). Baseline and peak cTnI levels within 48 h of PCI were normalized to the upper reference limit (URL). The URL for the cTnI assays was 0.04 ng/ml. The cTnI thresholds for PMI were adjusted according to the fourth universal definition of myocardial infarction (4th UDMI) and the Society for Cardiovascular Angiography and Interventions (2014) [SCAI (2014)] and Academic Research Consortium (ARC)-2 criteria.

### 2.4 Endpoints

The primary study outcomes were all-cause mortality and cardiovascular mortality. Deaths that could not be attributed to a non-cardiac etiology were considered to be cardiac. Some secondary endings beyond that are listed below. Myocardial infarction was defined according to the third edition of the UDMI [8]. Revascularization was defined as a repeat vascularization procedure for ischemic symptoms and events driven by PCI or surgery involving any vessel [9]. Bleeding was quantified according to the Bleeding Academic Research Consortium Definition criteria and included types 2, 3, and 5 [10]. Major adverse cardiovascular and cerebrovascular events were defined as death, myocardial infarction, need for revascularization, and stroke. All endpoints were adjudicated centrally by two independent cardiologists, and disagreements were resolved by consensus.

### 2.5 Follow-up

All patients were assessed at 6 months, 1 year, 2 years, and 5 years post-PCI by clinic visit or telephone. Patients were advised to return for coronary angiography if clinically indicated by symptoms or documentation of myocardial ischemia.

### 2.6 Statistical analysis

For baseline characteristics, continuous variables are summarized as the mean ± standard deviation and categorical variables as the percentage. After normalizing the post-procedural cTnI values to the URL, intervals of cTnI was referring to the commonly used threshold: <1 × URL; 1 to 5 × URL; 5 to 35 × URL; 35 to 70 × URL; and ≥70 × URL. Kaplan–Meier curves were constructed to show the time-to-first event of death from any cause or cardiovascular death according to cTnI group. Univariable and multivariable Cox proportional hazards regression models were used to examine the associations between cTnI groups and deaths from any cause or cardiovascular deaths and to identify the most effective post-procedural cTnI threshold. Tests for trend were performed across the different cTnI groups. Covariates with a *P*-value of <0.1 in univariable analysis were included in the multivariable Cox analysis. All *P*-values were two-tailed, and differences between groups were considered statistically significant at P < 0.05. Statistical analyses were performed using SPSS version R26.0.0.0.

## 3 Results

### 3.1 Study population

**Fig. 1:**
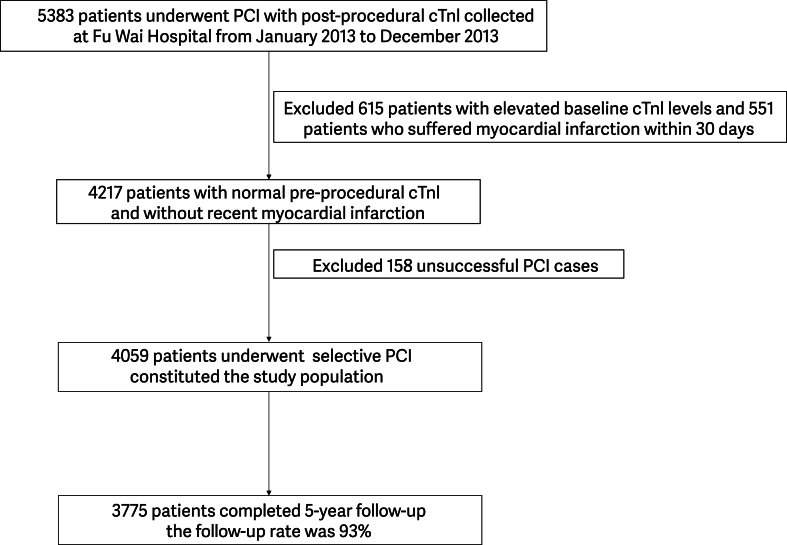
Flow chart.

During the study period, 5383 consecutive patients who underwent PCI at Fu Wai Hospital had post-procedural cTnI values available. After excluding 615 patients (11.4%) with elevated cTnI at baseline, 551 (10.2%) with myocardial infarction within 30 days of PCI, and 158 (2.9%) in whom PCI was unsuccessful, 4059 patients met the inclusion criteria and constituted the study population (Fig. 1). Baseline characteristics are shown according to cTnI level in Table 1. The characteristics of the entire study population are summarized in Supplemental Table 2. The mean patient age was 58.7 years and 74.5% were male. The mean BMI (calculated as kg/m^2^) was approximately 25.9, and the mean LVEF was 63.9%; 50.3% had unstable angina, 30.6% had diabetes mellitus, 65.3% had hypertension, and 67% had hyperlipidemia. The most common lesion was single-vessel disease (78.5%). The intravascular ultrasound use rate was 3.6%. The radial artery was used for vascular access in 91.3% of patients, and drug-eluting stents were implanted in 98% of cases. The mean SYNTAX score was 10.8 and the mean residual SYNTAX score was 7.7.

**Table 1:** Baseline characteristics according to cTnI threshold group.

	＜1 × URL	1-5 × URL	5-35 × URL	35-70 × URL	≥70 × URL
Age, y	57.6	58.2	60.1	61.2	61.5
Male	1004 (75%)	1026 (74.3%)	774 (72.2%)	105 (77.2%)	107 (81.1%)
BMI, kg/㎡	26.1	25.8	25.8	25.9	25.6
eGFR≤60 ml/min	40 (3.3%)	30 (2.3%)	43 (4.2%)	9 (7.4%)	9 (7.4%)
Diabetes mellitus	446 (33.3%)	409 (29.6%)	312 (29.1%)	49 (36%)	30 (22.7%)
Hypertension	849 (63.5%)	892 (64.6%)	714 (66.6%)	102 (75%)	93 (70.5%)
Hyperlipidemia	910 (68%)	903 (65.4%)	718 (67%)	92 (67.6%)	97 (73.5%)
Smoking history	752 (56.2%)	781 (56.6%)	563 (52.5%)	81 (59.6%)	79 (59.8%)
Prior PCI	305 (22.8%)	353 (25.6%)	257 (24%)	37 (27.2%)	35 (26.5%)
Prior MI	240 (17.9%)	291 (21.1%)	214 (20%)	36 (26.4%)	32 (24.2%)
Prior CVD	118 (8.8%)	139 (10.1%)	128 (11.9%)	30 (22.1%)	16 (12.1%)
Family history of CAD	336 (25.1%)	356 (25.8%)	258 (24.1%)	43 (31.6%)	28 (21.2%)
Baseline SYNTAX	9.3	10.8	12.1	13.2	14.6
Residual SYNTAX	6.4	7.7	8.9	8.6	11.3
Clinical diagnosis					
Unstable Angina	651 (53%)	642 (50%)	523 (51.4%)	49 (40.5%)	56 (45.9%)
Stable Angina	477 (38.8%)	524 (40.7%)	394 (38.8%)	51 (42.1%)	49 (40.2%)
Asymptomatic Myocardial Ischemia	100 (8.2%)	121 (9.3%)	100 (9.8%)	21 (17.4)	17 (13.9%)

BMI, body mass index; CAD, coronary artery disease; cTnI, cardiac troponin I; CVD, cerebrovascular disease; eGFR, estimated glomerular filtration rate; MI, myocardial infarction; PCI, percutaneous coronary intervention; URL, upper reference limit.

### 3.2 Biomarker levels

A cTnI ≥5 × URL by the 4th UDMI [11], ≥35 × URL by the ARC-2 [12], and ≥70 × URL by the SCAI (2014) [13] was found in 6.6% and 3.3% of patients, respectively. The populations exceeding the different thresholds are shown in Supplementary Table 3.

### 3.3 Clinical outcomes

**Fig. 2:**
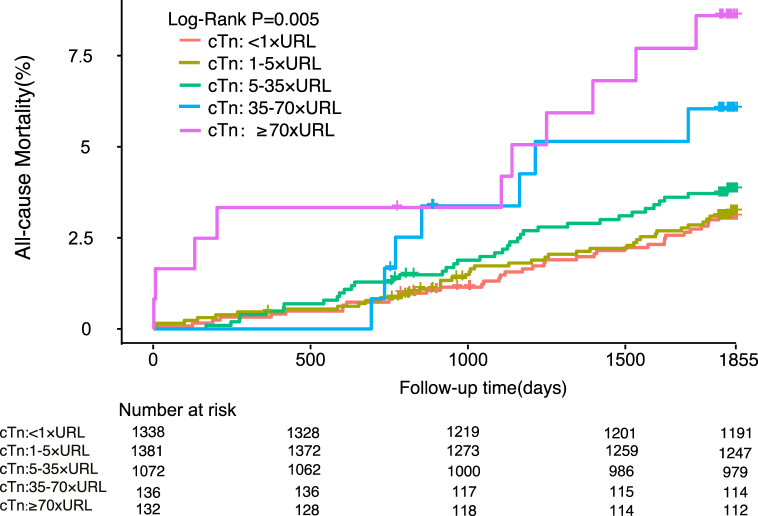
Kaplan-Meier curves depicting 5-year all-cause mortality based on post-percutaneous coronary intervention peak cTn levels categorized by various definitions.

**Fig. 3:**
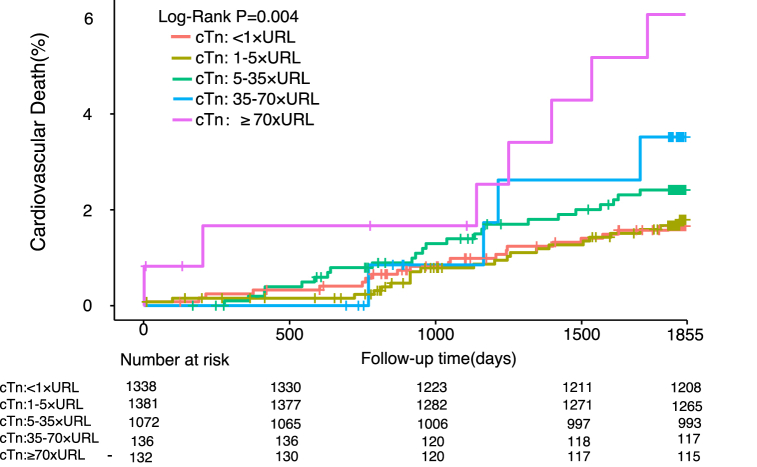
Kaplan-Meier curves depicting 5-year C based on post-percutaneous coronary intervention peak cTn levels categorized by various definitions.

In total, 3775 patients (93.0%) completed 5 years of follow-up; during this time, all-cause mortality was 3.4% (n = 132) and cardiovascular mortality was 2.0% (n = 77). The incidences of other clinical events during follow-up are shown in Supplemental Table 4. The number of adverse cardiovascular events increased cumulatively over time during medium-term to long-term follow-up, indicating that there are still many challenges to improvement of the prognosis after PCI. The incidences of important events are shown according to cTnI level in Table 2. Kaplan–Meier curves constructed after dividing the cTnI values into five groups (Fig. 2, Fig. 3) showed that the risk of death increased progressively as the post-procedural cTnI value increased from cTnI <1 × URL (all-cause mortality, 3.0%; cardiovascular mortality, 1.6%) to 1–5 × URL (all-cause mortality, 3.1%; cardiovascular mortality, 1.7%), 5–35 × URL (all-cause mortality, 3.7%; cardiovascular mortality, 2.4%); 35–70 × URL (all-cause mortality, 5.8%; cardiovascular mortality, 3.3%); and ≥70 × URL (all-cause mortality, 8.2%; cardiovascular mortality, 5.7%).

**Table 2:** Incidence of important clinical events according to cTnI threshold group.

**Clinical events**	**cTnI threshold**				
	＜1 × URL	1–5 × URL	5–35 × URL	35–70 × URL	≥70 × URL
All-cause Mortality	36 (2.7%)	39 (2.8%)	39 (3.6%)	7 (5.1%)	11 (8.3%)
Cardiovascular Death	20 (1.5%)	21 (1.5%)	24 (2.2%)	4 (2.9%)	8 (6.1%)
MI	61 (4.6%)	81 (5.9%)	65 (6.1%)	8 (5.9%)	11 (8.3%)
Revascularization	164 (12.3%)	187 (13.5%)	167 (15.6%)	22 (16.2%)	18 (13.6%)
Stroke	49 (3.7%)	41 (3%)	45 (4.2%)	5 (3.7%)	4 (3%)
Bleeding	168 (12.6%)	187 (13.5%)	157 (14.6%)	16 (11.8%)	17 (12.9%)
MACCE	262 (19.6%)	283 (20.5%)	250 (23.3%)	35 (25.7%)	31 (23.5%)

cTnI, cardiac troponin I; MACCE, major adverse cardiovascular and cerebrovascular events; MI, myocardial infarction; URL, upper reference limit.

**Fig. 4:**
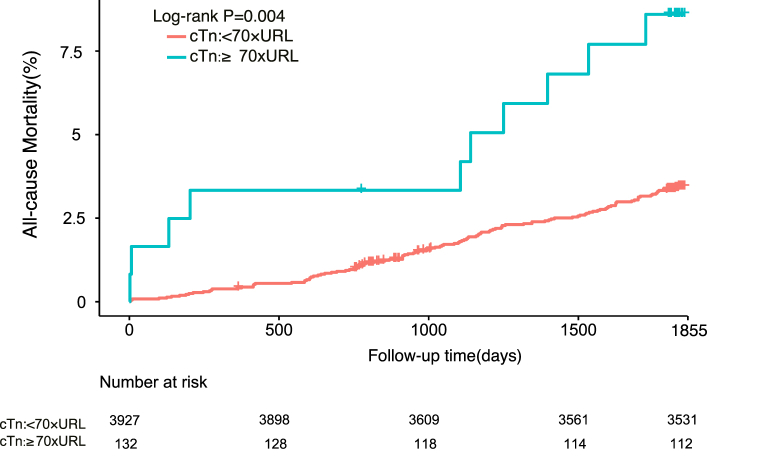
Kaplan-Meier curves of 5-year all-cause mortality based on the positive cTn threshold (≥70 × URL).

**Fig. 5:**
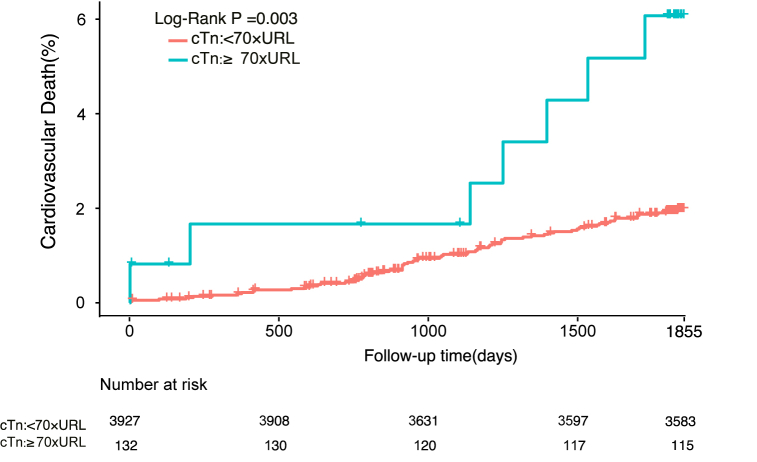
Kaplan-Meier curves of 5-year cardiovascular death based on the positive cTn threshold (≥70 × URL).

As shown in Table 3, both 5-year all-cause mortality and cardiovascular mortality were independently predicted by a cTnI value of ≥70 × URL (unadjusted hazard ratio [HR] 2.82, 95% confidence interval [CI] 1.40–5.66, P for trend 0.005 and unadjusted HR 3.65, 95% CI 1.54–8.62, P for trend 0.004, respectively). Addition of selected covariates (age, male sex, estimated glomerular filtration rate ≤60 ml/min, previous myocardial infarction, left ventricular ejection fraction <40%, drug-eluting stent placement, SYNTAX score, hypertension, and diabetes mellitus) to the model did not change the predictive significance of a cTnI level of ≥70 × URL in terms of 5-year all-cause mortality or cardiovascular mortality (adjusted HR 2.45, 95% CI 1.20–5.02, P for trend 0.032 and adjusted HR 3.17, 95% CI 1.31–7.67, P for trend 0.015, respectively). There was no statistically significant difference in all-cause or cardiovascular mortality for the other thresholds. For further confirmation, we divided the cTnI values into two groups based on the 70 × URL threshold (shown in Fig. 4, Fig. 5) and found significant differences in the Kaplan–Meier mortality curves: the all-cause and cardiovascular mortality rates were higher in patients with a cTnI level of ≥70 × URL (8.2% and 5.7%, respectively) than in those with a cTnI level of <70 × URL (3.3% and 1.9%). Multivariable analysis further supported this finding. As shown in Table 4, the SCAI (2014) threshold was significantly associated with 5-year cardiovascular mortality (adjusted HR 2.66, 95% CI 1.20–5.89; P = 0.016) and all-cause mortality (adjusted HR 2.23, 95% CI 1.16–4.30; P = 0.017). However, as described in Supplemental Table 5, the cTnI threshold did not seem to have a meaningful predictive ability in terms of other significant clinical events, which may have been influenced by other independent factors.

**Table 3:** Five-year all-cause mortality and cardiovascular mortality according to cTnI threshold group.

	**All-cause Mortality**		**Cardiovascular Death**	
	Unadjusted HR (95%CI)^a^	P	Unadjusted HR (95%CI)^a^	P
***cTnI:***				
＜1 × URL	*as the reference*			
1–5 × URL	1.04 (0.66–1.62)	0.87	1.05 (0.58–1.93)	0.87
5–35 × URL	1.26 (0.80–1.98)	0.32	1.47 (0.81–2.66)	0.21
35–70 × URL	1.95 (0.87–4.37)	0.11	2.06 (0.71–6.04)	0.19
≥70 × URL	2.82 (1.40–5.66)	0.004	3.65 (1.54–8.62)	0.003
	Adjusted HR (95%CI)^b^	P	Adjusted HR (95%CI)^b^	P
***cTnI:***				
＜1 × URL	*as the reference*			
1–5 × URL	1.04 (0.67–1.64)	0.86	1.08 (0.59–1.99)	0.81
5–35 × URL	1.18 (0.74–1.87)	0.49	1.44 (0.79–2.63)	0.24
35–70 × URL	1.53 (0.67–3.48)	0.31	1.59 (0.53–4.73)	0.41
≥70 × URL	2.45 (1.20–5.02)	0.014	3.17 (1.31–7.67)	0.01

The adjusted variables include age, male sex, estimated glomerular filtration rate ≤60 ml/min, prior myocardial infarction, left ventricular ejection fraction <40%, drug-eluting stent, SYNTAX score, hypertension, and diabetes mellitus.

a Unadjusted HR.

b Adjusted HR. CI, confidence interval; cTnI, cardiac troponin I; HR, hazard ratio; URL, upper reference limit.

**Table 4:** Results of multivariable adjusted analysis of 5-year all-cause mortality and cardiovascular mortality.

	**All-cause Mortality**		**Cardiovascular Death**	
	Adjusted HR (95%CI)^a^	P	Adjusted HR (95%CI)^a^	P
cTnI ≥ 70 × URL (reference: cTnI ＜70 × URL)	2.23 (1.16–4.30)	0.017	2.66 (1.20–5.89)	0.016
Age, years	1.06 (1.04–1.08)	<0.001	1.07 (1.04–1.09)	<0.001
Male	1.36 (0.90–2.05)	0.14	1.79 (1.00–3.20)	0.05
eGFR ≤ 60 ml/min (reference: eGFR ＞60 ml/min**)**	1.81 (1.03–3.20)	0.04	1.84 (0.90–3.73)	0.09
LVEF ＜ 40% (reference: LVEF ≥ 40%)	6.29 (2.77–14.27)	<0.001	6.58 (2.46–17.60)	<0.001
DES	0.70 (0.26–1.90)	0.48	0.42 (0.15–1.17)	0.10
DM	1.28 (0.90–1.83)	0.17	1.24 (0.78–1.98)	0.37
Hypertension	1.62 (1.07–2.46)	0.02	1.77 (1.02–3.09)	0.04
Prior MI	1.30 (0.86–1.96)	0.22	1.49 (0.89–2.51)	0.13
SYNTAX scores	0.98 (0.96–1.00)	0.09	0.97 (0.94–1.00)	0.03

a Adjusted HR. CI, confidence interval; DES, drug-eluting stent; DM, diabetes mellitus; eGFR, estimated glomerular filtration rate; LVEF, left ventricular ejection fraction; MI, myocardial infarction.

## 4 Discussion

In this large-scale study, we investigated the effect of different post-procedural cTnI thresholds (4th UDMI, ARC-2, and SCAI) on the long-term prognosis in 4059 patients with normal pre-procedural cTnI who underwent selective PCI at Fu Wai Hospital in 2013. The two main findings of the study were as follows: only the SCAI threshold (cTnI ≥70 × URL) was independently associated with an increased risk of 5-year all-cause mortality and cardiovascular mortality and 5-year all-cause and cardiovascular mortality gradually increased with higher post-procedural cTnI.

Post-procedural cTnI is the biomarker best able to represent the extent of myocardial damage. However, the current thresholds for post-procedural cTnI values that adversely affect the prognosis remain controversial. The most commonly used definitions are those in the 4th UDMI, ARC-2, and SCAI (2014), in which the respective cTn thresholds are ≥5 × URL, ≥35 × URL, and ≥70 × URL, respectively. Some centers have considered that a lower threshold is beneficial for reducing the number of false-negative errors and increasing the detection rate. A pooled analysis by Silvain et al. [14] found that a cTn of >5 × URL as proposed in the 4th UDMI was an independent predictor of 1-year all-cause mortality (adjusted odds ratio 3.21, 95% CI 1.42–7.27, P = 0.005) while a cTn >1 × URL was too sensitive to accurately predict mortality. However cTn >3 × URL could independently predict 1-year all-cause mortality, suggesting that a smaller cTn elevation was also associated with the prognosis [14]. However, other researchers felt that such a low standard was too sensitive to assess the clinical prognosis and preferred to use a higher threshold to identify patients with a poor prognosis. For example, the EXCEL study confirmed that a post-procedural CK-MB peak of ≥10 × URL was associated with cardiovascular mortality at 3 years and was an independent risk factor for 3-year all-cause mortality, whereas a lower degree of myocardial necrosis was not associated with a worse prognosis [15]. Xu et al. also showed that a high post-procedural CK-MB level (≥10 × URL) independently predicted 3-year cardiovascular and all-cause mortality [16], which was consistent with the findings of EXCEL study.

In our study, Cox proportional hazards regression analysis using a cTnI value of <1 × URL as the reference identified 1340 patients (33%) who exceeded the threshold set by the 4th UDMI (cTn ≥5 × URL), which was sensitive for detection of myocardial necrosis but had limited ability to predict the long-term prognosis. The difference was statistically significant only when cTnI was ≥70 × URL, which is consistent with the SCAI (2014) definition. Furthermore, as shown by the Kaplan–Meier curves, the post-procedural cTnI was positively correlated with all-cause mortality and cardiovascular mortality, both of which increased with a higher peak cTnI, suggesting that the more severe the myocardial necrosis, the worse the prognosis.

A study by Ueki et al. compared the diagnostic value of the different PMI definitions in 4404 patients with chronic coronary syndrome who underwent PCI. The incidence of PMI was lower with the ARC-2 and SCAI definitions than with the 3rd and 4th UDMI definitions. However, PMI as defined by ARC-2 (HR 3.90, 95% CI 1.54–9.93) and SCAI (HR 7.66, 95% CI 3.64–16.11) were better able to predict cardiovascular mortality at 1 year [17]. Consistent with their findings, we found that a cTnI ≥5 × URL did not affect the prognosis but that a cTnI ≥70 × URL was independently associated with long-term mortality, indicating the presence of massive myocardial necrosis. However, our study did not collect other evidence of ischemia, such as electrocardiographic changes, imaging features indicating new regional wall motion abnormalities, or clinical presentations, which contribute to more accurate identification of PMI [18,19]. Therefore, in the absence of other evidence of ischemia, the prognosis was significant only when the cTnI value was ≥70 × URL.

Considering that cTnI is a specific myocardial biomarker recommended by the guidelines and expert consensus for diagnosis of PMI, this study focused only on the influence of cTnI elevation on the long-term prognosis and did not explore the prognostic significance of different CK-MB thresholds. Some studies have found that post-procedural CK-MB elevation is an independent predictor of adverse outcomes and that post-procedural elevation of cTn has no prognostic significance [16,20,21]. However, in our study, the peak post-procedural cTnI threshold defined by SCAI (2014) was significantly associated with 5-year cardiovascular mortality (adjusted HR 2.66, 95% CI 1.20–5.89; P = 0.016) and all-cause mortality (adjusted HR 2.23, 95% CI 1.16–4.30; P = 0.017). This difference may reflect the different exclusion criteria used in the study populations. Therefore, a higher post-procedural peak cTnI (≥70 × URL) was also an important predictor of a worse prognosis in our study.

## 5 Limitation

This study has several limitations. First, although screening and a statistical analysis were performed, unmeasured confounders may preclude a definitive conclusion. Furthermore, while the cTnI level typically peaks within 8 h post-PCI and gradually declines towards normal within 48 h, the constraints of non-continuous measurements in the clinical setting could potentially have led to underestimation or mischaracterization of the true extent of post-PCI myocardial injury in certain cases. Second, our data were collected from a single center. Therefore, our findings may not be applicable to centers with limited experience of PCI and a small throughput of patients. Third, our study enrolled patients who underwent PCI at Fu Wai Hospital in 2013. Advances in PCI technology and medical therapy during the past decade may influence the results. What's more, despite the large number of patients enrolled, the number of deaths was small, which may have affected the relationship between cTnI elevation and postoperative mortality. Finally, as mentioned earlier, we focused on the degree of myocardial damage represented by the cTnI level but did not consider other evidence of myocardial ischemia, which may have influenced our results.

## 6 Conclusion

This study identified a post-procedural cTnI ≥70 × URL to be an independent predictor of 5-year all-cause and cardiovascular mortality in consecutive patients with normal pre-procedural cTnI who underwent elective PCI. We demonstrated that the SCAI (2014) post-procedural cTnI threshold was independently associated with long-term mortality and that the ARC-2 and 4th UDMI thresholds were not.

## Data availability statement

Due to the nature of this research, participants of this study did not agree for their data to be shared publicly, so supporting data is not available.

## Ethics statement

The Institutional Review Board approved the study protocol, which is consistent with the principles that have their origin in the Declaration of Helsinki, and all patients enrolled in the study signed an informed consent for long-term follow-up. The ethics approval number is NO.2021-1501, by the Notification of Ethics Committee of Fuwai Hospital.

## Funding

The study was supported by 10.13039/100014717National Natural Science Foundation for Young Scholars of China (81900323), the 10.13039/501100012166National Key Research and Development Program of China (2016YFC 1301300 & 2016YFC1301301), National Clinical Research Center for Cardiovascular Diseases, 10.13039/501100011635Fuwai Hospital, Chinese Academy of Medical Sciences (NCRC2020013), CAMS Innovation Fund for Medical Sciences (2020-I2M-C&T-B-049) and CAMS Innovation Fund for Medical Sciences (2023-I2M-C&T-B-061).

## CRediT authorship contribution statement

**Queyun Sun:** Formal analysis, Writing – original draft. **Pei Zhu:** Formal analysis. **Jingjing Xu:** Formal analysis. **Lin Jiang:** Formal analysis. **Yan Chen:** Formal analysis. **Xueyan Zhao:** Investigation, Project administration. **Lei Song:** Methodology, Supervision. **Yuejin Yang:** Supervision, Validation. **Runlin Gao:** Conceptualization, Visualization. **Bo Xu:** Resources, Validation, Visualization. **Jinqing Yuan:** Funding acquisition, Project administration, Supervision, Validation. **Ying Song:** Funding acquisition, Supervision, Visualization, Writing – review & editing.

## Declaration of competing interest

The authors declare that they have no known competing financial interests or personal relationships that could have appeared to influence the work reported in this paper.
