# Supplementary material for: Post-procedural elevated cardiac troponin I and the association with 5-year mortality in patients undergoing elective PCI
Source: Heliyon. 2024 Mar 22;10(7):e27979. doi: 10.1016/j.heliyon.2024.e27979 (PMC11002541; doi:10.1016/j.heliyon.2024.e27979)
Supplement: Multimedia component 1 [file mmc1.docx]

**Supplemental Table 1. Commonly used definitions of periprocedural myocardial infarction**

| **Definition** | **Peak of post-procedure cTn**  **threshold (within 48 hours)** | **Other conditions required for diagnosis** |
| --- | --- | --- |
| SCAI (2014) | cTn≥70×ULN |  |
|  | cTn≥35×ULN | new pathologic Q-waves in ≥ 2 contiguous leads  (or new persistent Left Bundle Branch Block) |
| ARC-2 | cTn≥35×ULN | Include at least one of the following conditions:  (1) New significant Q waves or equivalent  (2) Flow-limiting angiographic complications  (3) New substantial loss of myocardium on imaging |
| 4^th^ UDMI | cTn≥5×ULN | Include at least one of the following conditions:  (1) New ischaemic ECG changes or development of new pathological Q waves  (2) Imaging evidence of new loss of viable myocardium or new regional wall motion abnormality in a pattern consistent with an ischaemic aetiology  (3) Angiographic findings consistent with a periprocedural flow-limiting complication  (4) Post-mortem demonstration of a procedure-related thrombus in the culprit artery, or a macroscopically large circumscribed area of necrosis with or without intra-myocardial haemorrhage |

**Supplemental Table 2. Patient characteristics**

| Age, y | 58.7 ± 9.8 |
| --- | --- |
| Male | 74.5% (3024/4059) |
| BMI, kg/㎡ | 25.9 ± 3.2 |
| Diabetes mellitus | 30.6% (1242/4059) |
| Hypertension | 65.3% (2650/4059) |
| Hyperlipidemia | 67% (2719/4059) |
| Smoking history | 55.5% (2253/4059) |
| COPD | 2.8% (113/4059) |
| Peripheral vascular disease | 2.7% (109/4059) |
| Prior PCI | 24.4% (990/4059) |
| Prior CABG | 4% (162/4059) |
| Prior myocardial infarction | 20% (812/4059) |
| Prior cerebrovascular disease | 10.6% (430/4059) |
| Family history of CVD | 25.2% (1023/4059) |
| LVEF, % | 63.9 ± 6.6 |
| Clinical presentation |  |
| Asymptomatic ischemia | 9.6% (390/4059) |
| Stable angina | 38.8% (1575/4059) |
| Unstable angina | 50.3% (2041/4059) |
| Culprit artery |  |
| Left Main Artery and Three-vessel disease | 122(3.2%) |
| Two-vessel disease | 691(18.3%) |
| Single-vessel disease | 2962(78.5%) |
| Lesion types |  |
| De novo | 3585(95%) |
| Restenosis | 190(5%) |
| Lesion classification |  |
| Type A | 408(10.8%) |
| Type B1 | 696(18.4%) |
| Type B2 | 767(20.3%) |
| Type C | 1904(50.4%) |
| Lesion location |  |
| Ostium | 466(12.3%) |
| Shaft | 2686(71.2%) |
| Distal bifurcation | 623(16.5%) |
| Calcification |  |
| Mild | 1537(40.7%) |
| Moderate | 486(12.9%) |
| Severe | 104(2.8%) |
| Complete occlusion | 420(11.1%) |
| Thrombus-containing lesions | 30(0.8%) |
| Use of Aspirin | 99.9%(4057/4059) |
| Use of Ticagrelor | 0.1%(4/4059) |
| Use of Clopidogrel | 99.9% (4053/4059) |
| Use of LMWH | 59.1% (2399/4059) |
| Use of Stain | 95.9% (3893/4059) |
| IVUS guidance | 3.6% (146/4059) |
| Intubation way |  |
| By radial artery | 91.3% (3706/4059) |
| By femoral artery | 6.7% (272/4059) |
| By other intubation way | 2% (81/4059) |
| Stent implantation | 98.5% (3998/4059) |
| Type of stent |  |
| DES | 98% (3978/4059) |
| BMS | 0.6% (24/4059) |
| Total number of stents per patient | 1.9 ± 1.0 |
| Operation time per patient, min | 32.32±26.05 |
| Lesion length, mm | 28.1±17.6 |
| SYNTAX score |  |
| Low (-22) | 32.5% (1319/4059) |
| Intermediate (23-32) | 5.6% (227/4059) |
| High (33-) | 1% (41/4059) |
| Baseline SYNTAX | 10.8 ± 7.7 |
| Residual SYNTAX | 7.7 ± 6.2 |

BMI, body mass index; BMS, bare metal stent; CABG, coronary artery bypass grafting; COPD, chronic obstructive pulmonary disease; CVD, cardiovascular disease; DES, drug-eluting stent; IVUS, intravenous ultrasound; LAD, left anterior descending; LCX, left circumflex; LM, left main artery; LMWH, low molecular weight heparin; LVEF, left ventricular ejection fraction; PCI, percutaneous coronary intervention; RCA, right coronary artery

**Supplemental Table 3. Number of patients in each cTnI threshold**

| **cTnI threshold** | **Patients whose post-procedural cTnI exceeded the threshold (n：4059)** |
| --- | --- |
| cTnI＜1×URL | 1338 (33.0%) |
| cTnI≥1×URL | 2721 (67.0%) |
| cTnI≥5×URL (4^th^ UDMI threshold) | 1340 (33.0%) |
| cTnI≥35×URL (ARC-2 threshold) | 268 (6.6%) |
| cTnI≥70×URL (SCAI2014 threshold) | 132 (3.3%) |

4th UDMI, fourth edition of the universal definition of myocardial infarction; ARC-2, Academic Research Consortium-2; cTnI, cardiac troponin I; SCAI, Society for Cardiovascular Angiography and Interventions; URL, upper reference limit

**Supplemental Table 4. Cumulative rate of significant clinical events during follow-up**

| **Clinical events** | **Number of patients (n:3775)**  **6 months 1 year 2 years 5 years** | | | |
| --- | --- | --- | --- | --- |
| All-cause Mortality | 9 (0.2%) | 18 (0.4%) | 39(1%) | 132 (3.4%) |
| Cardiovascular Death | 4 (0.1%) | 9 (0.2%) | 20(0.5%) | 77 (2.0%) |
| MI | 55 (1.4%) | 61 (1.6%) | 75(2%) | 226 (5.9%) |
| Revascularization | 79 (2.0%) | 237 (6.2%) | 347(9.2%) | 558 (14.7%) |
| Stroke | 9 (0.2%) | 31 (0.8%) | 56(1.5%) | 144 (3.8%) |
| Bleeding | 61 (1.6%) | 184 (4.8%) | 253(6.7%) | 546 (14.4%) |
| MACCE | 137 (3.6%) | 326 (8.6%) | 477(12.6%) | 861 (22.8%) |

MACCE, major adverse cardiovascular and cerebrovascular events; MI, myocardial infarction

**Supplemental Table 5. Analysis of other significant clinical events by cTnI level**

|  | **Myocardial Infarction** |  | | **Revascularization** |  |
| --- | --- | --- | --- | --- | --- |
| ***cTnI:*** | **Unadjusted HR (95%CI)^a^** | | **P** | **Unadjusted HR (95%CI)^a^** | **P** |
| ＜1×URL | *as the reference* | | | |  |
| 1-5×URL | 1.28(0.92-1.79) | 0.15 | | 1.10(0.89-1.35） | 0.38 |
| 5-35×URL | 1.31(0.92-1.86) | 0.13 | | 1.27(1.02-1.57) | 0.03 |
| 35-70×URL | 1.33(0.64-2.78) | 0.45 | | 1.36(0.87-2.13) | 0.17 |
| ≥70×URL | 1.93(1.02-3.67) | 0.04 | | 1.15(0.71-1.87) | 0.57 |
|  | **Stroke** |  | | **Bleeding** |  |
| ***cTnI:*** | **Unadjusted HR (95%CI)^a^** | | **P** | **Unadjusted HR (95%CI)^a^** | **P** |
| ＜1×URL | *as the reference* | | | |  |
| 1-5×URL | 0.77(0.51-1.15) | 0.20 | | 1.00(0.82-1.22) | 0.98 |
| 5-35×URL | 1.07(0.71-1.59) | 0.75 | | 1.08(0.87-1.33) | 0.49 |
| 35-70×URL | 0.96(0.38-2.41) | 0.93 | | 0.88(0.54-1.45) | 0.62 |
| ≥70×URL | 0.80(0.29-2.22) | 0.67 | | 0.92(0.56-1.51) | 0.73 |
|  | **Major Adverse Cardiovascular and Cerebrovascular Events** | | | | |
| ***cTnI:*** | **Unadjusted HR (95%CI)^a.^ P** | | | | |
| ＜1×URL | *as the reference* | | | | |
| 1-5×URL | 1.05(0.89-1.24) 0.59 | | | | |
| 5-35×URL | 1.21(1.02-1.44) 0.03 | | | | |
| 35-70×URL | 1.35(0.95-1.92) 0.10 | | | | |
| ≥70×URL | 1.24(0.85-1.79) 0.27 | | | | |

CI, confidence interval; cTnI, cardiac troponin I; HR, hazard ratio; URL, upper reference limit
